# Sedimentary DNA and Fossil Archives Reveal Long‐Term Phytoplankton Shifts and Their Biogeochemical Influence in a Subtropical Lake

**DOI:** 10.1111/mec.70509

**Published:** 2026-08-17

**Authors:** Yu Zhao, Jianan Zheng, Meifang Zhong, Wenxiu Zheng, Bo Qin, Kexin Zhu, Chenxue Zhang, Yanjie Zhao, Enlou Zhang, Eric Capo, Rong Wang

**Affiliations:** ^1^ Key Laboratory of Lake and Watershed Science for Water Security, Nanjing Institute of Geography and Limnology Chinese Academy of Sciences Nanjing China; ^2^ Department of Ecology, Environment and Geoscience Umeå University Umeå Sweden; ^3^ School of Geography and Ocean Science Nanjing University Nanjing China; ^4^ College of Urban and Environmental Sciences Hubei Normal University Huangshi China; ^5^ University of Chinese Academy of Sciences Beijing China; ^6^ Poyang Lake Wetland Research Station, Nanjing Institute of Geography and Limnology Chinese Academy of Sciences Jiujiang China

**Keywords:** climate change, diatom, eDNA, eutrophication, regime shift

## Abstract

Understanding long‐term phytoplankton community dynamics is essential for assessing ecological resilience and informing lake management under global change. However, centennial‐scale patterns of phytoplankton dynamics remain poorly resolved due to the limited taxonomic resolution of traditional fossil‐based paleoecological approaches. In this study, a combination of sedimentary DNA (sedDNA) and subfossil diatom analysis was used to investigate changes in the phytoplankton community over the past two centuries and associated sedimentary biogeochemical responses in Lake Xiliang, a subtropical lake in the Yangtze River Basin, China. The sedDNA data captured major shifts in both eukaryotic and cyanobacterial communities, revealing distinct ecological phases linked to human activities and regional climate warming. Under near‐natural conditions with minimal human disturbance, phytoplankton communities typically exhibit low diversity and are characterized by oligotrophic taxa such as xanthophytes and *Gyrosigma*. After the 1930s, increases in epiphytic *Staurosira* and declines in tychoplanktonic *Aulacoseira* suggest reduced river–lake connectivity and weaker water‐column turbulence following the construction of the Jinshui Sluice, likely facilitating macrophyte expansion. In recent decades, intensified anthropogenic pressures combined with regional warming have driven a shift toward more diverse phytoplankton assemblages enriched in cyanobacteria, reflecting elevated nutrient inputs and rising temperatures. The observed associations between phytoplankton community changes and sedimentary geochemical proxies suggest that phytoplankton dynamics may not only have responded to environmental change but also contributed to sedimentary biogeochemical cycling, particularly total organic carbon accumulation and calcium‐related processes such as photosynthesis‐induced carbonate precipitation. These findings illustrate long‐term phytoplankton community dynamics and underscore the value of sedDNA for reconstructing ecological trajectories in lake ecosystems.

## 1 Introduction

Lakes are essential components of the Earth system, playing critical roles in the hydrological cycle, nutrient transport and climate regulation (Cole et al. 2007; Müller Schmied et al. 2014; Wohlfahrt et al. 2021). Over the past two centuries—particularly since the Industrial Revolution—lake ecosystems have undergone profound transformations due to accelerating anthropogenic pressures, including land use change, agricultural runoff and global warming. These factors have led to lake water quality deterioration, nutrient accumulation and loss of biodiversity (Brönmark and Hansson 2002; Anderson et al. 2012; Lin et al. 2022; Keck et al. 2025).

Phytoplankton, as the foundational primary producers in lake ecosystems, drive key ecological processes including primary production, nutrient cycling and carbon fixation (Carrick and Schelske 1997). Phytoplankton community dynamics are typically shaped by both biotic factors (e.g., competition) and abiotic factors (e.g., temperature and nutrient inputs), as noted by Reynolds (1987). Recent studies showed that 68% of lakes globally have experienced intensified summer algal blooms, highlighting a widespread decline in water quality (Ho et al. 2019). Because of their sensitivity to variations in temperature, light availability and nutrient inputs, phytoplankton communities respond rapidly to environmental changes, making them powerful indicators of ecosystem shifts (Paerl and Paul 2012). Understanding how phytoplankton communities respond to environmental change is essential for predicting future trajectories under ongoing anthropogenic and climatic pressures.

Most studies on phytoplankton community responses to environmental change rely on controlled experiments and ecological monitoring data. Manipulative experiments, which typically adjust key factors such as temperature and nutrients, have shown that warming can shift communities toward dominance by smaller taxa (e.g., cyanobacteria), potentially leading to algal blooms (Schindler 1974; Sommer et al. 2001). These studies, though valuable for identifying causal relationships, are often limited by simplified and short‐duration experimental settings, which cannot fully reflect complex natural processes. On the other hand, decades of monitoring data showed that lake ecosystems have shifted from macrophyte‐dominated to phytoplankton‐dominated stable states in regions with strong human disturbance (Smith 2003; Zhang et al. 2022). However, these monitoring records typically span only a few decades and rarely capture detailed information on phytoplankton community composition, especially in developing countries where long‐term monitoring programs were initiated relatively late. Yun et al. (2024) further emphasized that short‐term observations and ecological models often fail to capture the responses of organisms to climate and anthropogenic stressors.

Paleolimnological approaches provide powerful tools to overcome these limitations by reconstructing ecosystem histories over centuries to millennia, using biological and geochemical proxies preserved in lake sediments. Although these proxies—such as diatoms, cladocerans, chironomids and pollen grains—have provided reliable reconstructions of past ecological changes, they may not fully capture algal taxa that lack diagnostic physical subfossils in sediments, such as chlorophytes and cyanobacteria (Smol and Cumming 2000; Smol et al. 2001; Smol et al. 2004). This highlights the need to combine conventional paleolimnological techniques with emerging methods, such as sedimentary DNA (sedDNA) analysis (Capo et al. 2023). By extracting fragmented genetic material preserved in sediments, sedDNA enables high taxonomic resolution reconstruction of past biodiversity (Capo et al. 2021). Recent studies have shown that sedDNA provides powerful insights into the ways anthropogenic pressures have shaped freshwater ecosystems (Barouillet et al. 2023). In Sihailongwan Maar Lake, China, for example, a decline in phytoplankton diversity since the 1860s was attributed to intensified human influence (Yan et al. 2024). Likewise, Ibrahim et al. (2021) demonstrated that historical shifts in phytoplankton composition were linked to nutrient concentrations, highlighting the effectiveness of sedDNA in capturing diverse phytoplankton taxa across time.

Lake Xiliang, located in the middle and lower reaches of the Yangtze River, provides an ideal setting for investigating long‐term phytoplankton community dynamics and their environmental drivers. Over the past century, lakes in this region have experienced ecological transformations driven by intensified anthropogenic activities and climate warming, resulting in shifts in phytoplankton composition and loss of ecosystem services (Liu et al. 2013; Zhao et al. 2022). Century‐scale changes in non‐diatom phytoplankton groups remain poorly characterized, hindering a comprehensive understanding of phytoplankton community dynamics and lake ecosystem responses. In this study, we integrate traditional paleolimnological techniques with sedDNA analysis to reconstruct ~200 years of phytoplankton community (both diatom and non‐diatom) changes and associated geochemical changes in Lake Xiliang. We aimed to (1) reconstruct long‐term phytoplankton community dynamics in Lake Xiliang, (2) identify major phases of community change and their environmental drivers, and (3) evaluate the relationships between phytoplankton communities and sedimentary biogeochemical indicators.

## 2 Materials and Methods

### 2.1 Study Site

**FIGURE 1:**
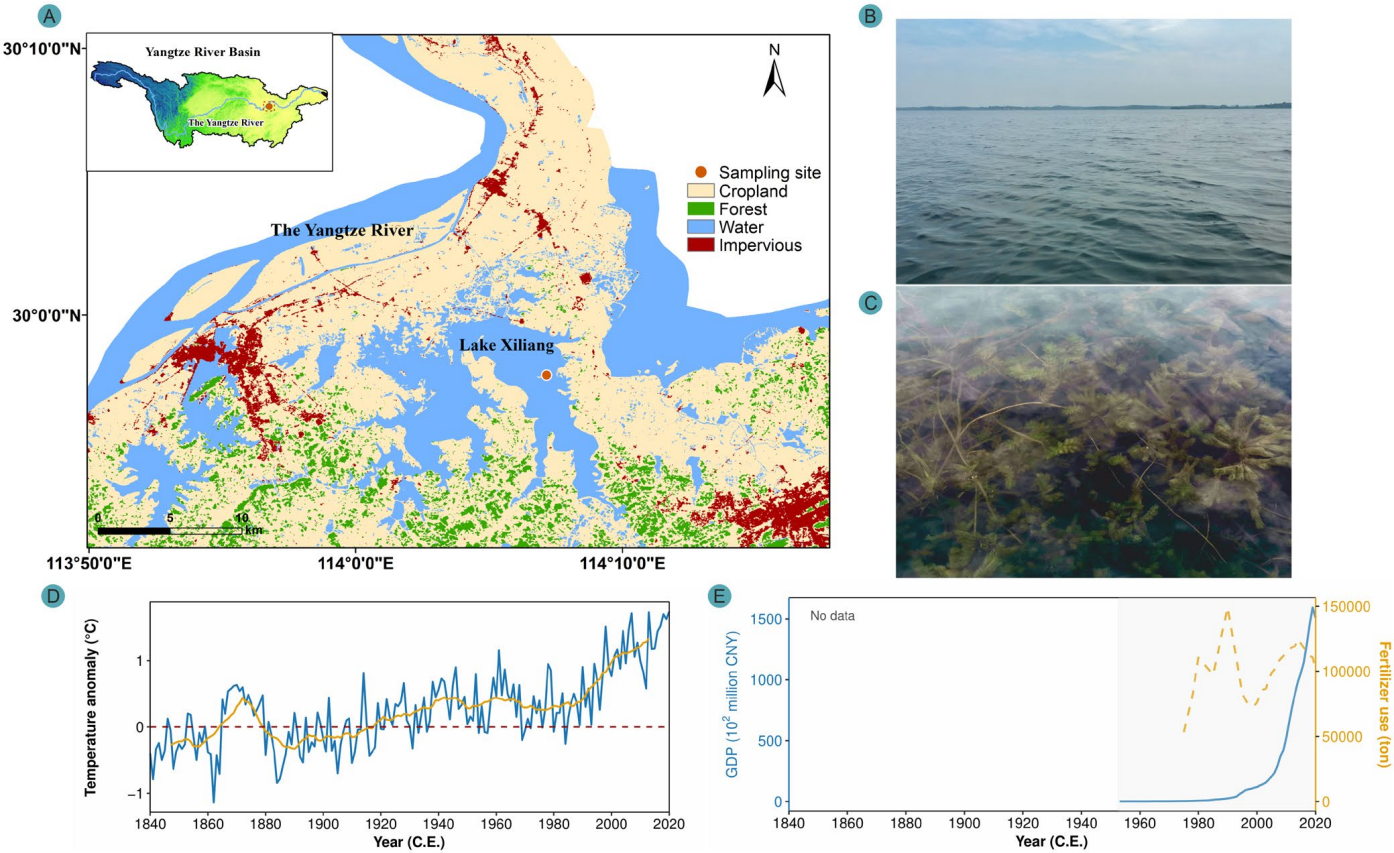
Lake location, temperature trend, socioeconomic development and current lake conditions of Lake Xiliang. (A) Location of Lake Xiliang, with surrounding land use types indicated (cropland, forest, impervious surfaces and water). The red dot marks the sampling site. The data are derived from remote sensing imagery collected in 2020. (B, C) Field photographs of Lake Xiliang taken during September 2019, showing shoreline vegetation. The images correspond to locations at 29°57′52.34″N, 114°06′52.95″ E and 29°57′54.88″N, 114°07′29.34″ E, respectively. All photographs were taken by Yu Zhao during sampling. (D) Regional temperature anomaly (°C) from 1840 to 2020. The orange line represents the moving average line. (E) Historical trends in gross domestic product (GDP) and fertilizer application in the study region. Gaps represent periods with unavailable data.

Lake Xiliang (29°57′46.01″N, 114°07′11.25″ E; 32 m a.s.l.; maximum depth: 4.1 m) is a shallow freshwater lake located in the middle and lower reaches of the Yangtze River, China (Wang and Dou 1998; Figure 1). The surrounding landscape is dominated by agricultural land, interspersed with patches of forest, grassland and impervious surfaces (Figure 1A). Prior to the 1930s, Lake Xiliang covered approximately 131.2 km^2^. Since the 1970s, extensive land reclamation around the shallow lake margins converted parts of the lake and adjacent littoral areas into agricultural or developed land, reducing the open‐water area to about 84 km^2^ (Cui et al. 2013; Hou et al. 2020). The region has a subtropical monsoon climate, characterized by high humidity, with an annual mean temperature of 16.8°C. In recent decades, the mean annual temperature has shown an increasing trend (Figure 1D). Since the 1980s, rapid economic development has been reflected by rising trends of gross domestic product (GDP) and fertilizer use (Figure 1E). Regional annual temperature data were obtained from the Berkeley Earth location‐based temperature record for the grid point nearest to Xianning City and Lake Xiliang (Rohde and Hausfather 2020). Historical GDP and fertilizer application data were obtained from the Xianning Statistical Yearbook (Xianning Municipal Bureau of Statistics and Survey Office of the National Bureau of Statistics in Xianning 2022). Field surveys conducted in 2019 revealed that certain areas of the lake still exhibit high water transparency and support abundant submerged macrophytes (Figure 1B,C).

### 2.2 Field Sampling and Subsampling

A 50 cm sediment core was retrieved from Lake Xiliang (29°57′46.0072″N, 114°7′11.2467″E; water depth: 3.5 m) using a gravity corer in 2020. The coring site was selected based on both its position within the central lake area and field‐measured water depth. Among several surveyed candidate locations, the selected site had the greatest measured depth, close to the reported maximum depth of approximately 4.1 m. The sediment core was immediately transported to the laboratory and stored at −20°C until processing. Subsampling for sedimentary DNA analysis was conducted in a dedicated clean DNA laboratory under sterile conditions. All tools and work surfaces were sterilized before use, and subsampling was performed using sterile disposable labware where possible and under a laminar flow hood to minimize contamination. The core was sliced at 1 cm intervals, yielding 50 subsamples. To reduce potential contamination from the outer core surface, only the central portion of each sediment slice was reserved for DNA extraction. The remaining material was freeze‐dried and used for radiometric dating, geochemical analyses and microscopic diatom analysis.

### 2.3 Chronology

The specific activities of ^210^Pb and ^137^Cs were measured using an HPGe GWL‐120‐15 detector (EG&G Ortec, USA). The chronological framework was established using the constant rate of supply model (CRS). Standard samples of ^137^Cs and ^226^Ra were provided by the China Institute of Atomic Energy, while ^210^Pb standard samples were verified by the University of Liverpool, UK, with a test error of less than 10%. Prior to analysis, the dating samples were dried and weighed. All analyses were conducted in the High Purity Germanium Well Gamma Meter Laboratory at Nantong University, China.

### 2.4 Geochemical Indicators

Total organic carbon (TOC), total nitrogen (TN), calcium (Ca), median grain size, magnetic susceptibility and phosphorus fractions were measured to infer long‐term variations in lake primary productivity, trophic status and sedimentary processes. TOC and TN measurements were conducted as follows: Approximately 0.5 g of milled sample was treated with 5% hydrochloric acid to remove inorganic carbonates. The sample was then repeatedly rinsed with distilled water until neutral pH was achieved and subsequently dried for analysis using an EA3000 Elemental Analyzer. Calcium (Ca) measurements were performed as follows: Approximately 0.15 g of dried sample was placed into a polytetrafluoroethylene (PTFE) digestion vessel. Then, 0.5 mL of hydrochloric acid, 6.0 mL of nitric acid and 3.0 mL of hydrofluoric acid were added. The mixture was digested using a Berghof MWS‐3 microwave digestion system at 180°C ± 5°C for 10–15 min. After cooling, 0.5 mL of perchloric acid was added, and the solution was evaporated at a medium temperature (180°C–200°C). Subsequently, 2.5 mL of 1 mol L^−1^ nitric acid, 0.25 mL of hydrogen peroxide and 5 mL of distilled water were added to dissolve the residue, followed by heating. After final cooling, the solution volume was adjusted to 25 mL. The resulting solution was stored at 4°C prior to analysis using inductively coupled plasma atomic emission spectrometry (ICP‐AES, USA). Grain size analysis was conducted with a Mastersizer 3000 laser particle size analyzer (Malvern Panalytical, UK) following pretreatment that removed organic matter with H_2_O_2_ and carbonates with dilute HCl. Magnetic susceptibility was measured using the MS2 magnetic susceptibility meter (Bartington Instruments Ltd., UK), and the final value was obtained as the average of three repeated measurements. Sediment phosphorus was fractionated into labile P (LP), Fe‐bound P (Fe‐P), Al‐bound P (Al‐P), Ca‐bound P (Ca‐P), organic P (Org‐P) and residual P (Res‐P) using a standard sequential extraction procedure (Yin et al. 2018). Phosphate concentrations in the extracts were measured using the UV‐2700 spectrophotometer (Shimadzu Corp., Japan).

### 2.5 Diatom Identification by Microscopic Analysis

Microscopic diatom analysis was conducted at an earlier stage of this study and focused on the upper 35 cm of the 50‐cm sediment core. This initial analysis was designed to examine recent diatom assemblage changes. During microscopic identification, diatom composition appeared relatively stable below approximately 22 cm; therefore, the initial diatom analysis was extended to 35 cm but was not continued to the bottom of the core. Subsequently, the sedDNA study was developed with a broader objective: to reconstruct longer‐term phytoplankton dynamics, including both diatom and non‐diatom groups, and to establish an earlier ecological baseline. Therefore, molecular and geochemical analyses were extended to the full 50 cm core, whereas microscopic diatom identification remained limited to the upper 35 cm.

Laboratory analysis of diatom samples was conducted following the protocols outlined by Battarbee et al. (2001). Initially, 0.2 g of freeze‐dried sediment was used for each diatom sample, to which 10% HCl and 30% hydrogen peroxide were added. The mixture was heated to eliminate carbonates and metal salt oxides until no further large bubbles were observed. Subsequently, the sample underwent centrifugation at 3000 r min^−1^ for 3 min, followed by decanting of the supernatant. This process was repeated three times. Diatoms were then identified and counted under an OLYMPUS BX‐53 microscope at a magnification of 10 × 100. A minimum of 500 valves were counted per sample. The identification of diatoms was primarily carried out according to Krammer and Lange‐Bertalot (Krammer and Lange‐Bertalot 1986; Krammer and Lange‐Bertalot 1988; Krammer and Lange‐Bertalot 1991a; Krammer and Lange‐Bertalot 1991b).

### 2.6 Molecular Analysis

The molecular analyses were carried out in a dedicated laboratory, following strict precautions to ensure the authenticity of the results. For each sample, DNA was extracted by using the ALFA‐SEQ Advanced Soil DNA Kit (mCHIP, Guangzhou, China) according to the manufacturer's instructions. For eukaryotic phytoplankton community, the V4 region of the 18S rRNA gene was amplified using primers 528F (5′‐GCGGTAATTCCAGCTCCAA‐3′) and 706R (5′‐AATCCRAGAATTTCACCTCT‐3′) (Elwood et al. 1985); for prokaryotic phytoplankton community, the V4–V5 region of the 16S rRNA gene was amplified using primers 515F (5′‐GTGCCAGCMGCCGCGGTAA‐3′) and 926R (5′‐CCGTCAATTCMTTTRAGTTT‐3′) (Xiao et al. 2017). Primers were synthesized by Invitrogen (Invitrogen, Carlsbad, CA, USA). PCR reactions were prepared with a total volume of 50 μL, containing 25 μL of 2× Premix Taq (Takara Biotechnology, Dalian Co. Ltd., China), 1 μL of each primer (10 μM), and 3 μL of DNA template (20 ng μL^−1^). The thermocycling conditions were as follows: initial denaturation at 94°C for 5 min, followed by 30 cycles of 30 s at 94°C for denaturation, 30 s at 52°C for annealing and 30 s at 72°C for extension, with a final elongation step at 72°C for 10 min. The PCR amplification was performed using a BioRad S1000 thermocycler (Bio‐Rad Laboratory, CA, USA). The length and concentration of the PCR products were checked using 1% agarose gel electrophoresis. The pooled PCR products were then purified with the E.Z.N.A. Gel Extraction Kit (Omega Bio‐tek, USA). Sequencing libraries were prepared using the NEBNext Ultra II DNA Library Prep Kit for Illumina (New England Biolabs, MA, USA), following the manufacturer's protocols, with index codes added during this step. Library quality and concentration were assessed using a Qubit 2.0 Fluorometer (Thermo Fisher Scientific, MA, USA). Finally, the libraries were sequenced on an Illumina Nova6000 platform (Guangdong Magigene Biotechnology Co. Ltd., Guangzhou, China).

### 2.7 Bioinformatics

Raw sequence reads were demultiplexed by the sequencing provider (Guangdong Magigene Biotechnology Co. Ltd.) using unique barcode sequences. Primers were trimmed off using Cutadapt (Martin 2011), and all subsequent analyses were performed in R (v4.2.1) using the DADA2 pipeline (Callahan et al. 2016; R Core Team 2020). Quality profiles of forward and reverse reads were inspected using plotQualityProfile(). Reads were filtered and trimmed with filterAndTrim() using parameters: truncLen = c(200, 150), maxEE = c(2, 2), truncQ = 2 and rm.phix = TRUE. After dereplication, error rates were modelled using learnErrors(), and denoising was performed with dada(). Paired‐end reads were merged using mergePairs(), and amplicon sequence variants (ASVs) were constructed with makeSequenceTable(). Chimeric sequences were identified and removed using removeBimeraDenovo() with the consensus method. Taxonomic assignment of non‐chimeric ASVs was conducted using the SILVA reference database (v132) via assignTaxonomy() (Callahan et al. 2016). The resulting ASV abundance tables and taxonomic annotations were exported for downstream analysis.

For the 18S metabarcoding data, a rarefaction curve was generated from the raw sequence dataset to evaluate whether sequencing depth was sufficient across all samples (Figure S1). Only ASVs assigned to Bacillariophyta (diatoms), Chlorophyta (chlorophytes), Chrysophyta (chrysophytes), Cryptophyta (cryptophytes), Dinoflagellata (dinoflagellates), Glaucophyta (glaucophytes) and Xanthophyta (xanthophytes) were retained for subsequent analyses. To standardize sequencing depth for comparative purposes, rarefaction was then performed using the rrarefy() function in the vegan package (Oksanen et al. 2009), based on the minimum sample read count (6186). For the 16S rRNA gene dataset, only ASVs annotated as cyanobacteria were retained for analysis. A total of 2883 reads corresponding to 39 cyanobacterial ASVs were obtained. Due to their relatively low abundance within total prokaryotic reads, rarefaction was not applied to preserve informative sequences. The final ASV table was subsequently used for downstream diversity and community structure analyses. To reduce high‐frequency noise and improve stratigraphic comparability, only the cyanobacterial ASV abundance data were averaged within 2‐cm depth bins. The resulting binned cyanobacterial abundance matrix was then plotted against depth.

### 2.8 Data Analysis

All statistical analyses and data visualization were performed in R. Differences in phosphorus fractions among time periods were assessed using one‐way ANOVA, followed by Tukey's HSD post hoc tests for pairwise comparisons, with significance set at *p* < 0.05.

For diatom fossil assemblages, constrained incremental sum of squares clustering (CONISS) was applied to define zonation and identify key transition boundaries (Grimm 1987). To compare the temporal trends of diatom genera derived from different approaches, smoothed curves were fitted using generalized additive models (GAMs) to highlight long‐term changes (Hastie 2017). The relationship between community dissimilarity and temporal distance was assessed using Mantel tests (Mantel 1967). The Mantel statistic (*r*) represents the Spearman correlation coefficient between two distance matrices: a Bray–Curtis dissimilarity matrix of community composition and a Euclidean distance matrix of sampling times. The significance of this correlation (*p*‐value) was determined through 999 permutations, indicating whether the observed association deviates significantly from random expectation. A larger Mantel *r* value indicates that communities become more different as the time interval between samples increases. ASV richness was used as a proxy for phytoplankton community diversity. The ASV richness change rate between two adjacent samples was calculated using Equation (1) to quantify community dynamics. Principal component analysis (PCA) was performed to summarize major gradients in eukaryotic phytoplankton community structure (Nichols 1977). The first principal component axis score (PCA1) was used to represent the primary variation pattern in community composition over time. Prior to Pearson correlation analysis, the data normality was tested using the Shapiro–Wilk test.

(1) $$\text{Richness change rate}=\frac{R_{i}-R_{i-1}}{t_{i}-t_{i-1}}$$

where richness change rate represents the temporal rate of ASV richness change between two adjacent samples, *R*_*i*_ and *R*_*i*−1_ are ASV richness values of adjacent samples, and *t*_*i*_ and *t*_*i*−1_ are their corresponding ages. Higher values indicate more rapid temporal changes in richness.

To assess associations between phytoplankton community and sedimentary biogeochemical indicators, we compiled a candidate set of 13 environmental variables, including TOC, TN, Ca, LP, C/N ratio, Fe‐P, Al‐P, Ca‐P, Org‐P, Res‐P, magnetic susceptibility, median grain size and temperature. Phytoplankton community variation was represented by ASV richness derived from sedimentary DNA, which has been shown to provide a useful molecular proxy for reconstructing past community changes in lake ecosystems (Anslan et al. 2022; Nwosu et al. 2023; Thorpe et al. 2022). We first screened the candidate variables for associations with phytoplankton richness using Spearman correlations and year‐residual Spearman correlations, the latter calculated after removing chronological trends from both variables. To address collinearity among the core biogeochemical variables TOC, TN, Ca and LP, principal component analysis (PCA) was performed, and the first principal component (BG‐PC1) was used as a composite index of sedimentary biogeochemical variation.

Redundancy analysis (RDA) was then used to quantify the association between phytoplankton richness and the environmental‐geochemical matrix. Models were fitted for both the full set of 13 candidate variables and the BG subset comprising TOC, TN, Ca and LP. Partial RDA included Year as a conditioning variable to evaluate whether phytoplankton–environment associations remained significant after controlling for chronological trends. Temporal Z‐score trends of richness and BG‐PC1 were calculated to visualize their synchronized changes over the past two centuries. Year‐residual Spearman correlations between BG‐PC1 and phylum‐level richness were further calculated to evaluate taxon‐specific contributions to biogeochemical changes. Spearman's rank correlation coefficient is denoted as rho. Multiple testing corrections were applied using the false discovery rate (FDR) method.

## 3 Results

### 3.1 Chronology and Geochemical Indicators

The 50 cm sediment core recovered from Lake Xiliang spans the period from 1827 to 2020 ce, capturing nearly 200 years (Figure S2). The chronology was established using the CRS model and independently checked against the ^137^Cs profile. Excess ^210^Pb activity generally decreased with depth, while a distinct ^137^Cs peak occurred at 15 cm, corresponding to 1963 ce in the CRS chronology and consistent with the 1963 global fallout maximum.

**FIGURE 2:**
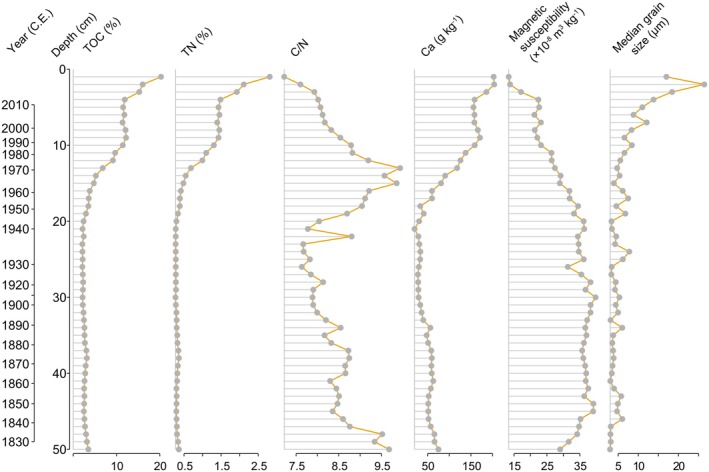
Temporal variations of total organic carbon (TOC), total nitrogen (TN), C/N ratio, calcium (Ca), magnetic susceptibility and median grain size across different sediment depths.

Before the 1940s, sedimentary TOC, TN and median grain size remained relatively stable, while the C/N ratio showed a gradual decline (Figure 2). Beginning in the 1940s, TOC and Ca contents increased, whereas magnetic susceptibility exhibited a concurrent decreasing trend. The C/N ratio rose from the 1940s and declined from the 1970s onward, whereas median grain size increased after the 1970s.

**FIGURE 3:**
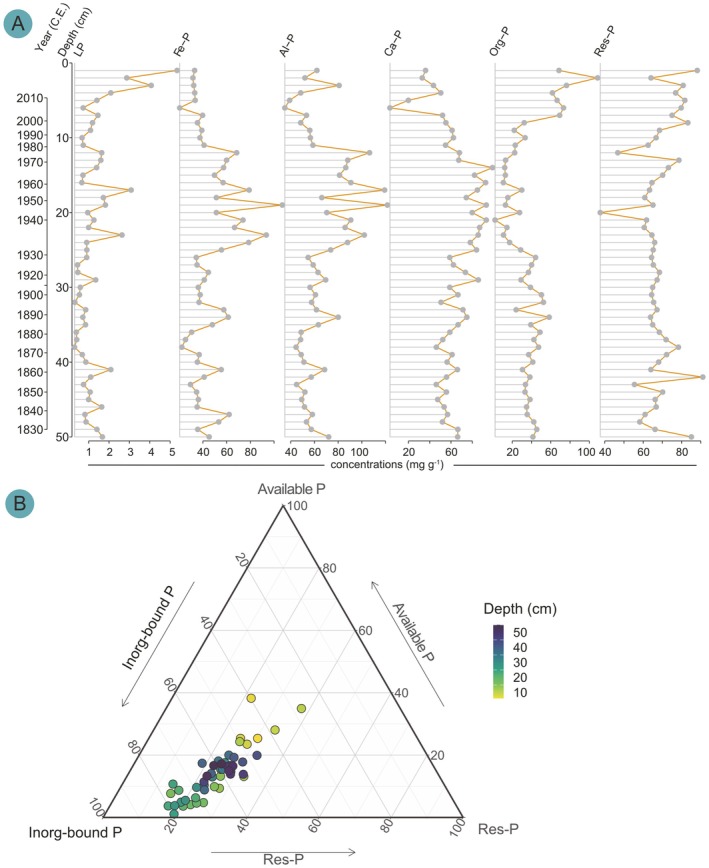
Phosphorus fractionation in the sediment core from Lake Xiliang. (A) Distribution of different phosphorus fractions, including labile phosphorus (LP), iron‐bound phosphorus (Fe‐P), aluminium‐bound phosphorus (Al‐P), calcium‐bound phosphorus (Ca‐P) and organic phosphorus (Org‐P). (B) Ternary diagram illustrating the relative abundance of available phosphorus (LP + Org‐P), inorganic‐bound phosphorus (Fe‐*P* + Al‐*P* + Ca‐P) and residual phosphorus (Res‐P) across sediment depths.

Different phosphorus fractions in the sediment exhibited distinct temporal variation patterns (Figure 3A). The concentration of labile phosphorus (LP) remained low overall but showed an increasing trend in the surface layers. Iron‐bound (Fe‐P), aluminium‐bound (Al‐P) and calcium‐bound phosphorus (Ca‐P) were significantly higher between the 1930s and 1970s compared with the pre‐1930s and post‐1970s periods (*p* < 0.05). Conversely, organic phosphorus (Org‐P) concentrations were relatively low between the 1930s and 1970s, followed by an increase in subsequent decades. The relative proportions of different phosphorus forms were visualized (Figure 3B). The composition was dominated by inorg‐bound P (Fe‐*P* + Al‐P + Ca‐P), with most samples clustering toward the inorg‐bound P apex. Residual phosphorus (Res‐P) constituted a moderate proportion, while available phosphorus (LP + Org‐P) generally exhibited the lowest relative proportion among the three fractions. Samples from shallower depths (lighter colours) tended to exhibit higher proportions of available P, whereas deeper samples (darker colours) shifted toward higher contributions of inorg‐bound P.

### 3.2 Diatom Assemblages Identified by Microscopy

**FIGURE 4:**
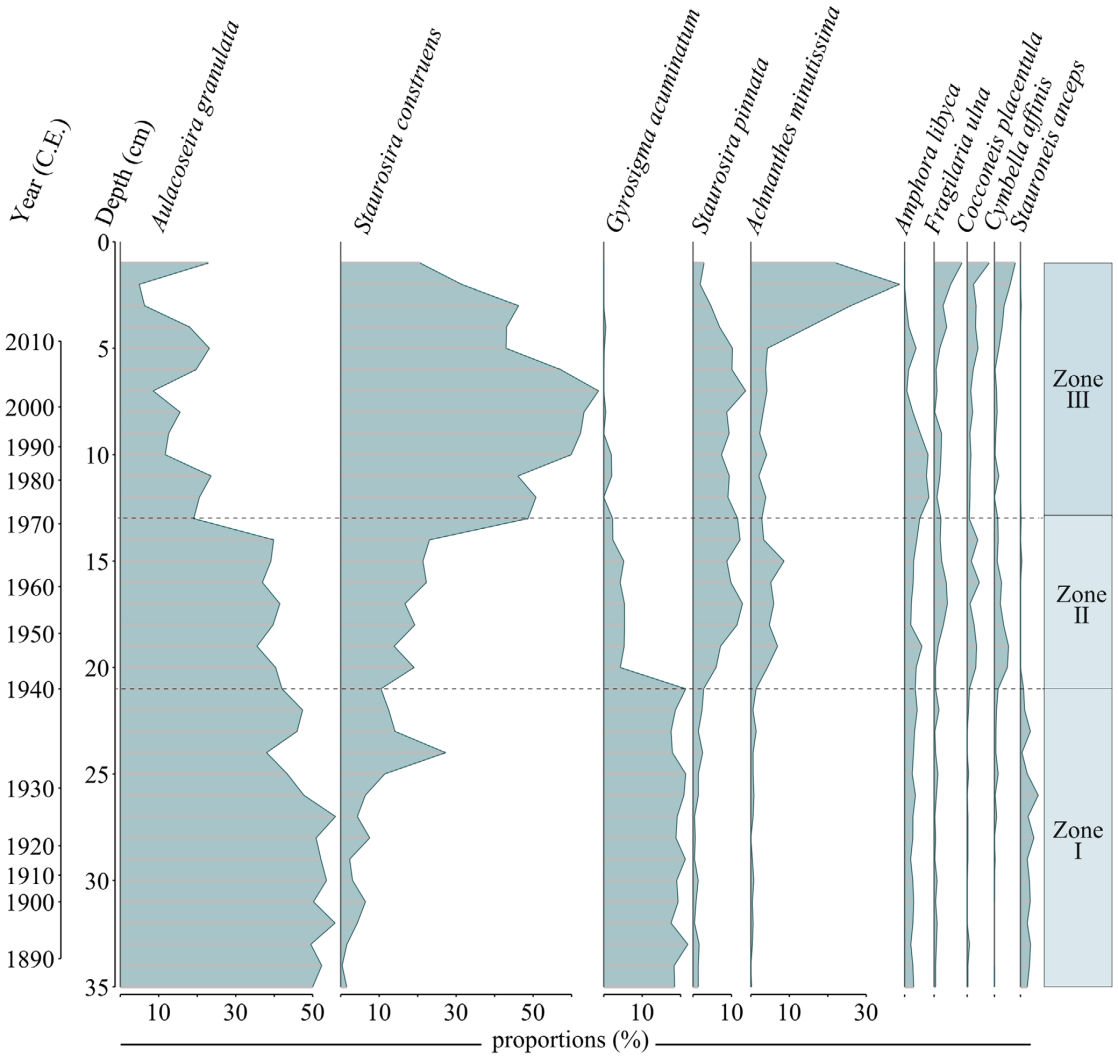
Changes in proportions of the main diatom species found in Lake Xiliang. Zonation was determined by CONISS (constrained incremental sum of squares) clustering.

Diatom zonation analysis identified the 1940s and 1970s as key transitional boundaries (Figure 4 and Figure S3). Throughout the analysed interval, *Aulacoseira* (mainly *Aulacoseira granulata* ) and *Staurosira* (mainly *Staurosira construens* ) consistently appeared in higher proportions compared to other genera (Figure 4). The proportion of *Aulacoseira* declined after the 1940s, from an average of 49.48% before the 1940s—significantly higher than the 24.83% observed thereafter (*p* < 0.05; Figure S3). After the 1970s, *Staurosira* showed a marked increase relative to earlier periods, although its proportion began to decline after the 2000s. In contrast, *Achnanthes minutissima* increased markedly after the 2000s, with an average proportion approximately 6 times higher than in earlier periods. *Gyrosigma*, mainly represented by *Gyrosigma acuminatum* , was more abundant before the 1940s but declined thereafter.

### 3.3 Changes in Phytoplankton Community Composition

**FIGURE 5:**
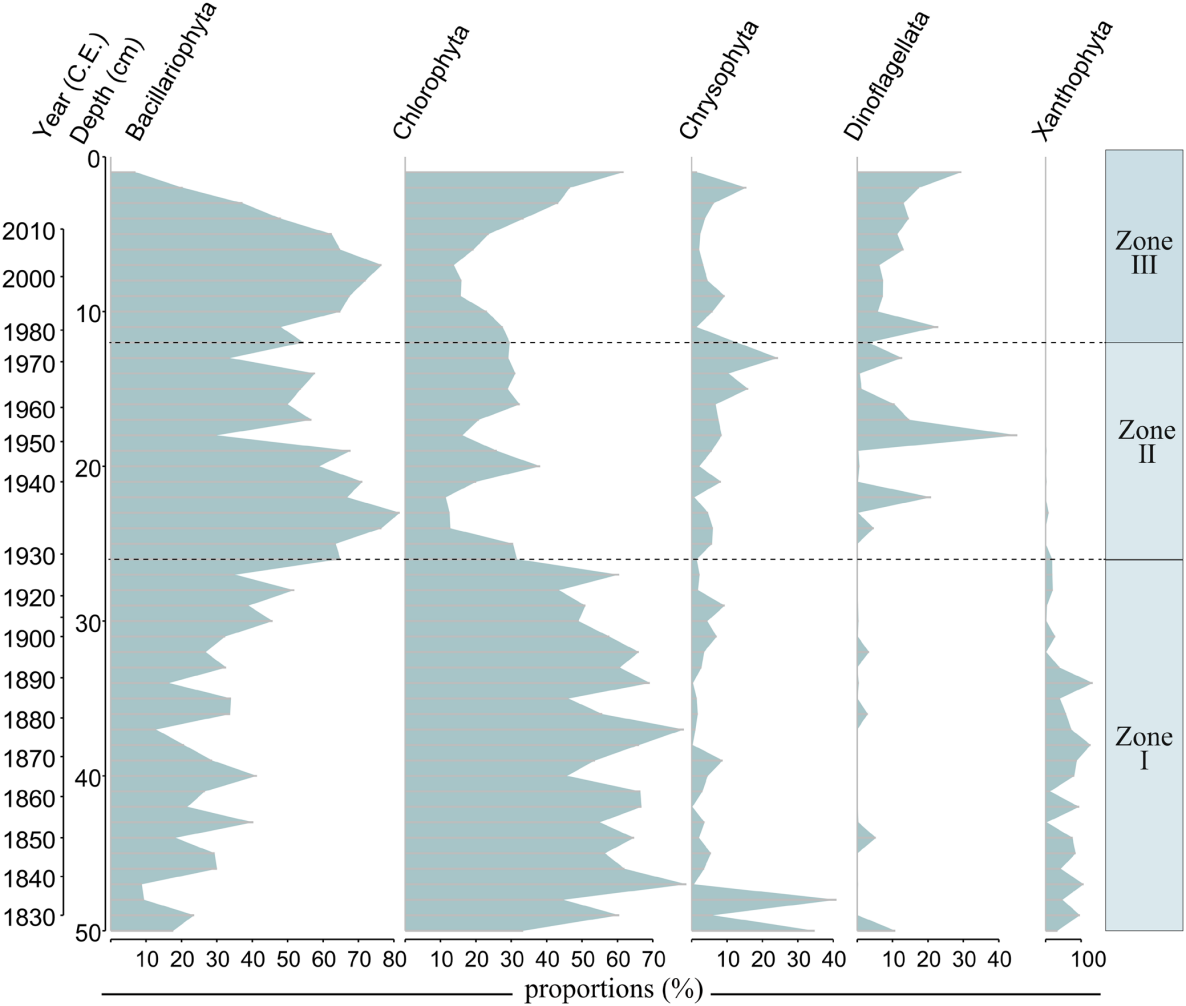
Proportions of major eukaryotic phytoplankton taxa in Lake Xiliang over the past two centuries, based on sedDNA data. Cryptophyta and Glaucophyta, with average relative abundances of less than 1%, are not shown.

A total of 291 ASVs were identified as members of the eukaryotic phytoplankton, encompassing the phyla Bacillariophyta (diatoms), Chlorophyta (chlorophytes), Chrysophyta (chrysophytes), Cryptophyta (cryptophytes), Dinoflagellata (dinoflagellates) and Xanthophyta (xanthophytes). SedDNA reconstruction revealed that diatoms and chlorophytes consistently maintained relatively high proportions over the historical period (Figure 5A). Cluster analysis identified three major phases in the historical dynamics of the eukaryotic phytoplankton community, with transition points occurring around the 1930s and 1970s. The proportion of diatoms was higher between the 1930s and 1970s, averaging 58.07%, compared to other periods. In contrast, chlorophytes showed lower proportions during the same interval, with an average of 24.49%. Chrysophytes did not exhibit a distinct temporal trend. In contrast, the proportions of dinoflagellates and xanthophytes varied significantly across time. Dinoflagellates became more abundant after the 1930s, with an average proportion of 1.18%, while xanthophytes were more dominant before the 1930s, averaging 5.32% compared to just 0.10% afterward. SedDNA reconstruction also recovered multiple diatom genera, and the temporal trends of the two dominant genera, *Aulacoseira* and *Staurosira*, were broadly consistent with those inferred from fossil analysis (Figure S4).

**FIGURE 6:**
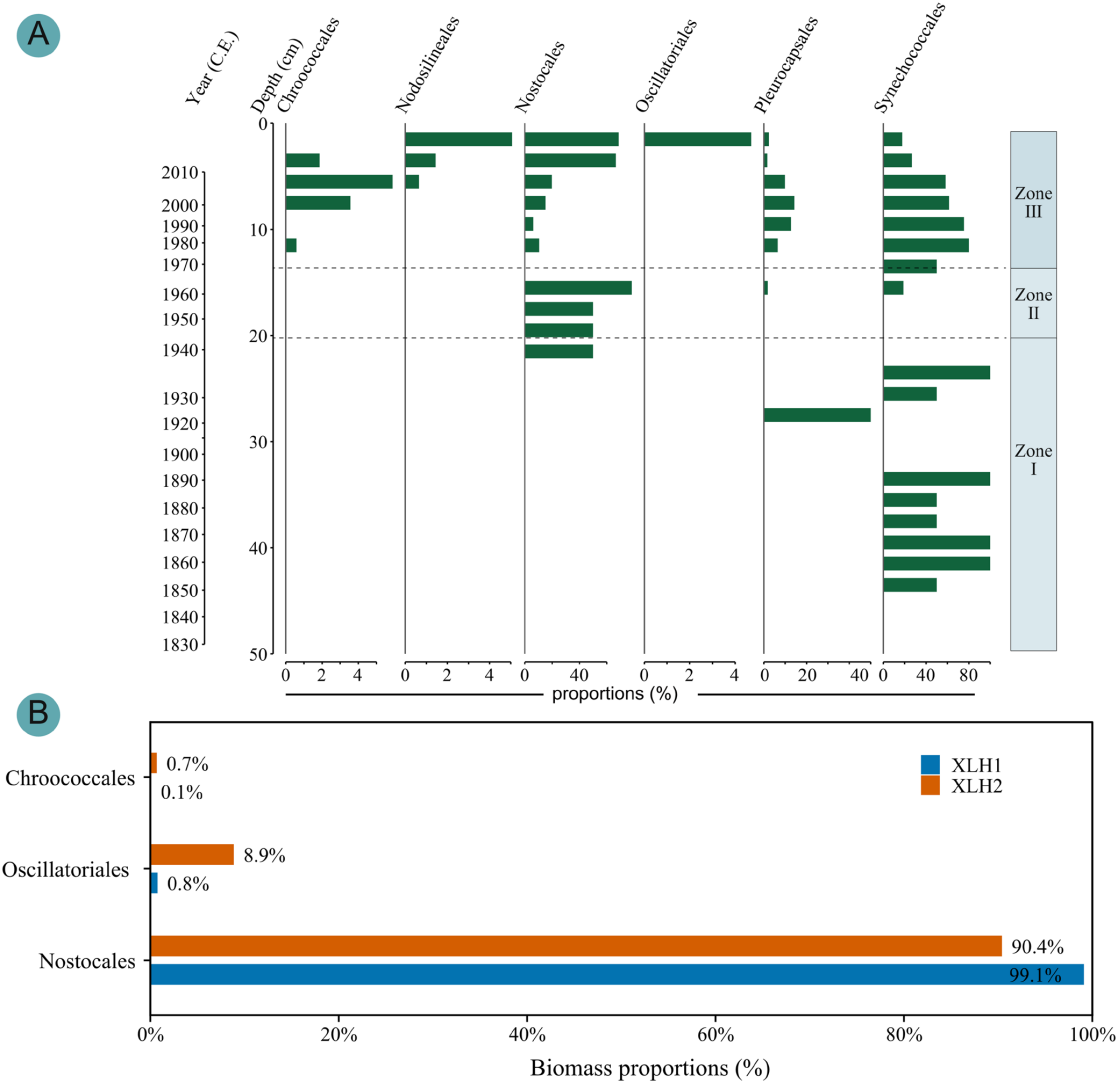
Proportions of cyanobacterial orders in Lake Xiliang based on sedimentary DNA data (A), and cyanobacterial biomass composition at the order level from two field sampling sites during the summer of 2020 (B). XLH1 was located at 29°56′40.13″N, 114°07′34.32″E, with a water depth of 2.3 m and a Secchi depth of 0.53 m; XLH2 was located at 29°56′36.46″N, 114°04′10.20″E, with a water depth of 2.7 m and a Secchi depth of 0.52 m.

In the prokaryotic dataset, 39 ASVs were identified as cyanobacteria. The proportions of cyanobacterial orders exhibited clear temporal variations throughout the sediment core (Figure 6A). Before the 1970s, the cyanobacterial community was relatively simple in composition, with Synechococcales and Nostocales comprising the majority of the assemblage. Oscillatoriales and Nodosilineales were only detected in the most recent sediment layers and were present in very low proportions. A marked increase in the proportions of Nostocales was observed after 2009. Additionally, Chroococcales and Pleurocapsales became more detectable after the 1970s.

A total of three cyanobacterial orders were identified in the field survey at the two sites (XLH1 and XLH2), fewer than the number detected in the sedimentary DNA record. Nostocales dominated both sites, comprising 99.1% and 90.4% of total cyanobacterial biomass, respectively, while Oscillatoriales and Chroococcales occurred at much lower proportions (Figure 6B).

### 3.4 Changes in Phytoplankton Community Diversity and Structure Over the Past Two Centuries

**FIGURE 7:**
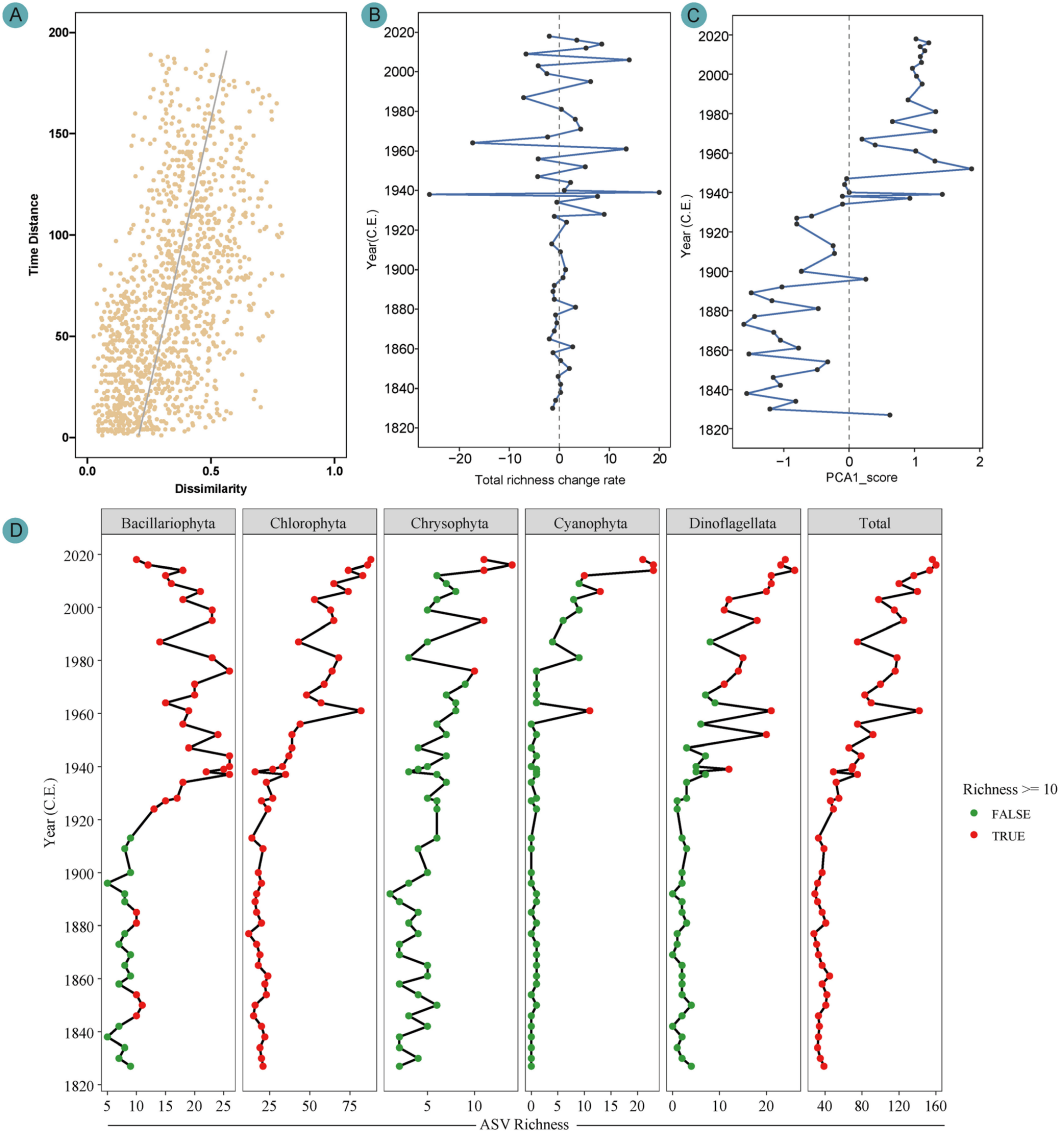
Changes in the phytoplankton community. (A) Relationship between eukaryotic phytoplankton community dissimilarity (Bray–Curtis) and temporal distance. (B) Temporal rates of change in total ASV richness of the eukaryotic phytoplankton community. (C) First principal component axis scores (PCA1), reflecting shifts in eukaryotic phytoplankton community. (D) Vertical profiles of ASV richness for major phytoplankton taxa (selected based on a mean ASV richness ≥ 2 across all samples) and for the total phytoplankton community over the past 200 years. Red points indicate ASV richness values greater than or equal to 10, whereas green points indicate values less than 10.

The eukaryotic phytoplankton community structure in Lake Xiliang underwent substantial changes over the past two centuries (Figure 7). Bray–Curtis dissimilarity values increased significantly with temporal distance (*r* = 0.547, *p* < 0.001; Figure 7A), indicating marked temporal changes in eukaryotic phytoplankton community structure over the past 200 years. Diatoms, chlorophytes, dinoflagellates, xanthophytes and cyanobacteria have also undergone substantial changes over the past two centuries, whereas chrysophytes show no clear trend (Figure S5).

The temporal change rate of overall phytoplankton community ASV richness was used to reflect community dynamics over the past 200 years (Figure 7B). Before the 1930s, the rate of community change was minimal (mean absolute value: 1.4), but it increased significantly after the 1930s (mean absolute value: 7.2), indicating structural shifts began during this period. Further, PCA axis 1 scores of the eukaryotic phytoplankton community also revealed structural changes after the 1930s (Figure 7C).

The ASV richness of different phyla and the entire phytoplankton community remained relatively stable before the 1930s (Figure 7D). Starting in the 1930s, total ASV richness showed a significant increasing trend. Notably, since the 1970s, cyanobacterial ASV richness began to increase from very low levels, while chlorophytes showed a similar upward trend; in contrast, diatoms showed a decreasing trend. The relationship between temperature and cyanobacterial ASV richness was analysed, revealing a significant positive correlation between temperature and diversity (*r* = 0.762, *p* < 0.001).

### 3.5 Relationships Between Phytoplankton Community and Biogeochemical Indicators

Residual correlation and partial RDA analyses revealed that phytoplankton ASV richness was significantly associated with sedimentary environmental variables. In the full model including all 13 candidate variables, phytoplankton ASV richness explained a significant proportion of environmental‐geochemical variation (*R* ^2^ = 0.339). This relationship remained significant after controlling for chronological trends (*p* = 0.0003). The BG subset, comprising TOC, TN, Ca and LP, showed stronger explanatory power (*R* ^2^ = 0.585). These results indicated that the BG subset captured the main sedimentary biogeochemical gradient associated with phytoplankton ASV richness.

**FIGURE 8:**
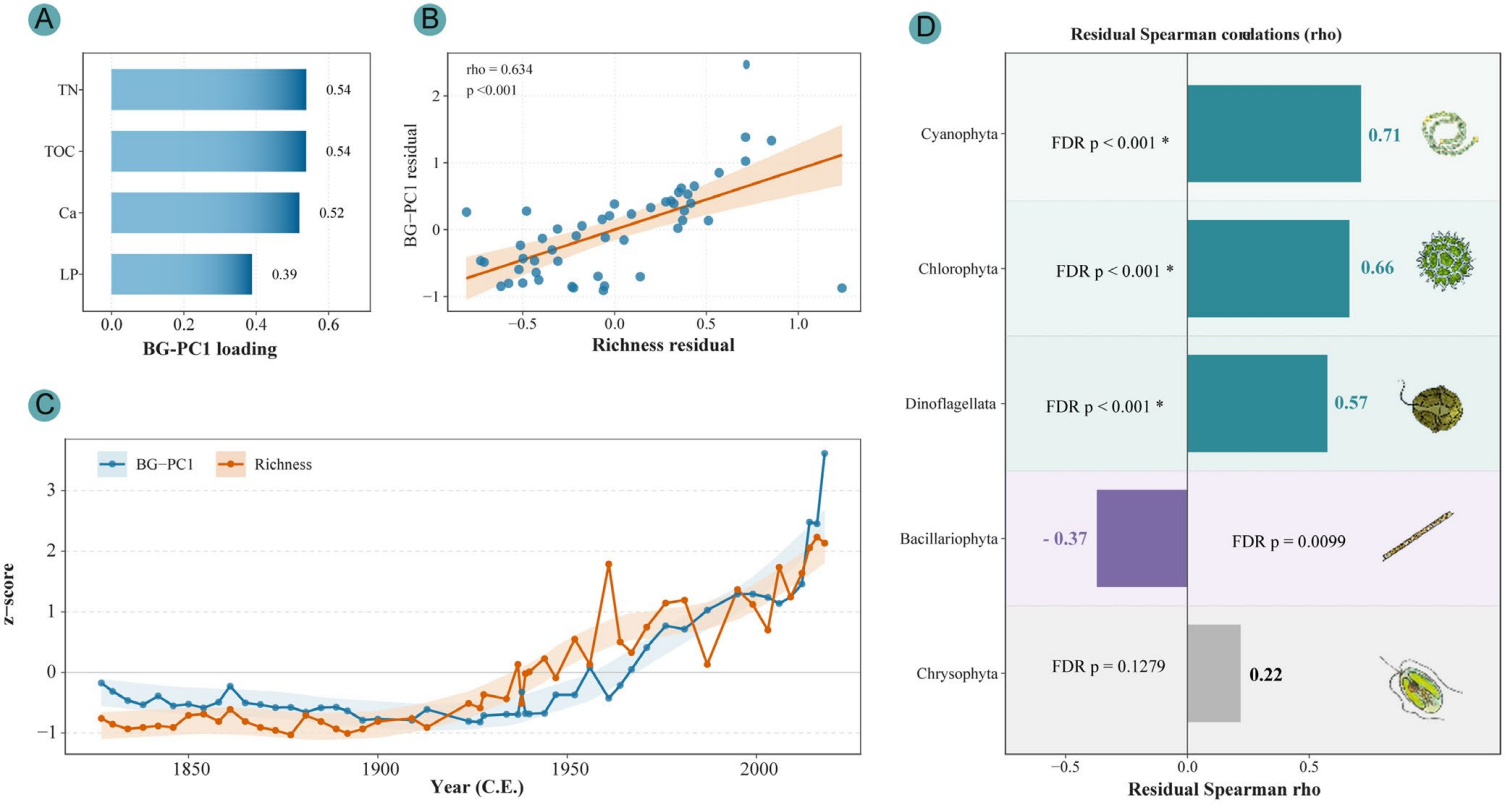
Relationships between phytoplankton community (ASV richness) and sedimentary geochemical indicators in Lake Xiliang. (A) Loadings of TOC, TN, Ca and LP on the first principal component (BG‐PC1). (B) Residual correlation between phytoplankton community and BG‐PC1 after controlling for chronological trends. (C) Temporal Z‐score trends of phytoplankton community and BG‐PC1. (D) Residual Spearman correlations between BG‐PC1 and phylum‐level phytoplankton. Numbers beside the bars indicate the corresponding correlation coefficients (rho), and FDR *p* values are shown for each group. Significant correlations are indicated by asterisks.

The first principal component of TOC, TN, Ca and LP (BG‐PC1) was calculated to represent the combined variation of these biogeochemical indicators and explained 84% of the variance (Figure 8A). Residual correlation analysis controlling for chronological trends showed a strong positive association between phytoplankton community and BG‐PC1 (rho = 0.634, *p* < 0.001; Figure 8B).

Temporal *Z*‐score trends demonstrated highly synchronized changes in phytoplankton ASV richness and BG‐PC1 over the past two centuries (Figure 8C). At the phylum level, Cyanophyta (rho = 0.71), chlorophytes (rho = 0.66) and dinoflagellates (rho = 0.57) were positively correlated with BG‐PC1, whereas diatoms showed a negative correlation (rho = −0.37), and chrysophytes were not significant (rho = 0.22; Figure 8D).

## 4 Discussion

This study revealed shifts in phytoplankton community composition in a lake located in the middle and lower reaches of the Yangtze River, spanning from the pre‐disturbance period to recent decades (Figures 4, 5, 6, 7). The observed phytoplankton community shifts were closely associated with hydrological engineering, intensified anthropogenic activities and regional climate warming. Furthermore, changes in phytoplankton communities were associated with variations in biogeochemical processes, highlighting their potential role in regulating lake ecosystem functioning. By integrating sedimentary DNA analysis with traditional paleoecological and geochemical records, this study enables a more comprehensive reconstruction of long‐term phytoplankton dynamics and associated lake ecosystem changes.

### 4.1 Phytoplankton Community Dynamics and Their Environmental Drivers Over the Past 200 Years

Phytoplankton community changes in Lake Xiliang were closely associated with changing environmental conditions over the past two centuries. Prior to the 1930s, the lake was oligotrophic with low phytoplankton diversity, low nutrient concentrations and dominance of oligotrophic taxa such as xanthophytes and *Gyrosigma acuminatum* , indicative of well‐mixed, high‐transparency conditions (Figures 2, 3 and 5; Pan et al. 2022). The co‐occurrence of benthic *Gyrosigma* and tychoplanktonic *Aulacoseira* likely reflects spatial and seasonal heterogeneity, with pelagic and benthic processes coexisting (Figure 4 and Figure S3; Li et al. 2026).

A pronounced shift in the eukaryotic phytoplankton community occurred after the 1930s (Figures 5 and 7), whereas cyanobacterial diversity remained low and compositionally simple (Figure 6). Diatoms increased in both proportions and diversity, while chlorophytes declined (Figure 5). Within the diatom assemblage, the epiphytic *Staurosira* increased markedly, whereas the tychoplanktonic *Aulacoseira*, typically favoured by water‐column turbulence, declined (Figure 4 and Figure S3). This turnover likely reflects weakened hydrodynamic disturbance caused by reduced river–lake connectivity after the construction of the Jinshui Sluice. Hydrological stabilization may have further promoted macrophyte expansion, providing substrates for epiphytic taxa such as *S. construens* (Blindow et al. 1993; Coops and Hosper 2002). Sedimentary proxies support this inference of enhanced macrophyte development after the 1930s. The moderate increase in C/N ratio, remaining below 15, suggests a greater contribution of aquatic macrophyte‐derived organic matter rather than dominant terrestrial inputs (Figure 2; Prahl et al. 1980; Ishiwatari and Uzaki 1987; Contreras et al. 2018). Elevated sedimentary Ca further supports this interpretation because macrophyte photosynthesis can increase pH and carbonate ion concentrations, promoting biogenic CaCO_3_ precipitation (Riding 2000; Liu et al. 2023; Zúñiga‐Barra et al. 2024), as also reported in Lake Fuxian (Du et al. 2025). This pattern is consistent with macrophyte expansion under stabilized hydrological regimes in other Yangtze River lakes after the 1950s (Huang et al. 2021). During this time period, stabilizing feedbacks—such as nutrient uptake by macrophytes and habitat provisioning for zooplankton grazers—likely contributed to maintaining phytoplankton biomass and a clear‐water state (Perrow et al. 1999; Burks et al. 2006).

In recent decades, nutrient enrichment and climate warming have broadly promoted eutrophication and phytoplankton community shifts in lake ecosystems (Paerl 2017; Paerl et al. 2018). In Lake Xiliang, increasing TOC and available phosphorus concentrations since the 1970s likely reflect enhanced nutrient loading and primary productivity (Figure 2). During this period, phytoplankton ASV richness increased, and the eukaryotic community showed higher richness change rates and larger PCA1 fluctuations than during the pre‐1930s period (Figure 7B,C), indicating increased community instability. This shift was marked by declines in diatom relative abundance and diversity. At the same time, oligotrophic taxa such as xanthophytes became scarce, while cyanobacterial diversity and dinoflagellate proportions increased, indicating a transition toward more nutrient‐rich conditions (Figures 5, 6, 7). Meanwhile, increased median grain size and higher *Staurosira* proportions after the 1970s may indicate stronger hydrodynamic disturbance, possibly related to lake‐area reduction and land‐use change (Figure 2 and Figure S3; Hou et al. 2020).

Changes in cyanobacteria composition at the order level further support this transition (Monchamp and Pick 2023). The higher proportion of Synechococcales before the 1970s likely reflected their advantage under oligotrophic conditions, particularly their high affinity for orthophosphate uptake (Moutin et al. 2002; Domaizon et al. 2013; Figure 6). After the 1970s, Synechococcales declined while Nostocales increased, consistent with the ecological advantages of Nostocales under eutrophic but potentially nitrogen‐limited conditions, including complex morphology and nitrogen fixation (Stockner et al. 2000; Callieri and Stockner 2002). This transition is consistent with recent observations of a shift from Synechococcales toward bloom‐forming and potentially toxic Nostocales (Monchamp and Pick 2023), and with our monitoring records showing higher Nostocales contributions in modern phytoplankton assemblages (Figure 6B). The concurrent decline in sedimentary C/N ratios indicates an increased contribution of algal‐derived organic matter, suggesting enhanced algal production. This increase may have suppressed submerged macrophytes through light limitation and resource competition (van Nes et al. 2002; Zhao et al. 2024), consistent with the reported post‐1980s decline in macrophyte coverage and diversity in Lake Xiliang (Peng et al. 2003).

### 4.2 Effects of Phytoplankton Community Changes on Sedimentary Biogeochemical Processes

Phytoplankton communities are not only sensitive indicators of environmental change but also active regulators of lake biogeochemical cycling. In our study, changes in phytoplankton communities were closely associated with sedimentary environmental variations. After controlling for chronological trends, seven candidate environmental variables were significantly correlated with phytoplankton ASV richness. The BG subset, including TOC, TN, Ca and LP, showed the strongest relationship with richness, suggesting that phytoplankton community was closely associated with sedimentary biogeochemical processes. Diatoms were negatively correlated with BG‐PC1, in contrast to cyanobacteria, chlorophytes and dinoflagellates, suggesting a reduced relative contribution of diatoms under nutrient‐rich conditions (Figure 7D).

This association between phytoplankton community change and sedimentary biogeochemical variation may partly arise from biological processes within the lake. Phytoplankton biomass deposition directly contributes to sedimentary TOC, while photosynthesis‐induced CO_2_ uptake increases pH and carbonate ion concentrations, thereby promoting carbonate precipitation (Du et al. 2025). The resulting accumulation of organic matter and biogenic carbonate can dilute magnetic minerals and reduce magnetic susceptibility (Zhang et al. 2012). Meanwhile, carbonate precipitation may promote phosphate co‐precipitation and the formation of relatively stable Ca–P compounds (Wang et al. 2022). In addition, macrophyte‐associated microbial consortia may enhance organic phosphorus mineralization, helping to explain the post‐1930s decline in organic P and the concurrent enrichment of mineral‐bound P (Belova 1993; Rejmánková and Sirová 2007). Nevertheless, this close association may also reflect shared responses to external forcing; for example, both phytoplankton and TOC can respond to warming and nutrient enrichment. By contrast, the Ca signal is more likely to reflect internal biological processes, as Lake Xiliang is not located in a karst region and no substantial external Ca source has been documented. Taken together, these patterns suggest that sedimentary biogeochemical processes were shaped by interacting external drivers and in‐lake biological processes.

Under continued eutrophic conditions, organic matter accumulation and decomposition at the sediment–water interface can lead to hypoxia or anoxia, promoting the release of previously immobilized Fe‐ and Ca‐bound phosphorus (Hupfer and Lewandowski 2008). In Lake Xiliang, the recent rise in sedimentary available P indicates an enlarged labile P pool, which may favour cyanobacterial dominance and delay macrophyte recovery after external inputs decline. This highlights a legacy effect of eutrophication and a persistent restoration challenge in eutrophic lakes of the middle and lower reaches of Yangtze River.

### 4.3 Implications

Multiple proxies revealed that phytoplankton communities in Lake Xiliang responded markedly to hydrological modification, eutrophication and warming, with corresponding changes in sedimentary carbon and calcium dynamics. Continued climate warming and eutrophication may reinforce cyanobacterial dominance and hinder macrophyte recovery, highlighting the need to consider internal nutrient loading and shifts in phytoplankton community composition alongside external nutrient reduction. Although sedDNA provides a powerful tool for tracking multi‐group community change, its interpretation should be integrated with conventional paleolimnological proxies to account for potential taphonomic and molecular biases.

## Author Contributions

Conceptualisation: Y.Z., J.Z., R.W., E.C. and E.Z. Developing methods: Y.Z., J.Z., M.Z. and E.C. Conducting the research: Y.Z., W.Z., B.Q. and C.Z. Data analysis, data interpretation, preparation of figures and tables: Y.Z., E.C., M.Z., K.Z. and Y.j.Z. All authors contributed to the writing and revision of the manuscript.

## Funding

This work was supported by the National Key Research and Development Program of China (2022YFF0801100) and the National Natural Science Foundation of China (42507663, 42507606, and 42307561). R.W. acknowledges the support of the NERC RESTORE project (NE/X014150/1). E.Z. acknowledges the financial support of the Double Thousand Plan of Jiangxi Province (jxsq2023101035).

## Ethics Statement

Ethical approval was not required because this study involved only environmental sediment and water samples. All samples were collected in accordance with relevant local regulations.

## Conflicts of Interest

The authors declare no conflicts of interest.

## Supporting information


**Figure S1:** Rarefaction curves of eukaryotic phytoplankton community in Lake Xiliang, depicting observed ASVs in response to sequencing depth.
**Figure S2:** Vertical profiles of ^210^Pb and ^137^Cs activities, with a distinct ^137^Cs peak at 1963, and the age‐depth model based on the CRS method.
**Figure S3:** Changes in proportions of the main diatom genera (> 3%) found in Lake Xiliang. Zonation was determined by CONISS (constrained incremental sum of squares) clustering.
**Figure S4:** Comparison of diatom reconstructions from sedimentary DNA and subfossil records. Smoothed trends were fitted using generalized additive models (GAMs). Diatom fossil data included only samples from the 1–35 cm interval.
**Figure S5:** Relationship between phytoplankton community dissimilarity (Bray–Curtis) and temporal distance.

## Data Availability

The data that support the findings of this study are openly available in Dryad at https://doi.org/10.5061/dryad.mw6m906cj.
