# Supplementary material for: Sedimentary DNA and Fossil Archives Reveal Long‐Term Phytoplankton Shifts and Their Biogeochemical Influence in a Subtropical Lake
Source: Mol Ecol. 2026 Aug 17;35(16):e70509. doi: 10.1111/mec.70509 (PMC13480152; doi:10.1111/mec.70509)
Supplement: Supplementary file 1 [file MEC-35-e70509-s001.docx]

**Supplementary materials**

Figure S1. Rarefaction curves of eukaryotic phytoplankton community in Lake Xiliang, depicting observed ASVs in response to sequencing depth.

Figure S2. Vertical profiles of ^210^Pb and ^137^Cs activities, with a distinct ^137^Cs peak at 1963, and the age-depth model based on the CRS method.

Figure S3. Changes in proportions of the main diatom genera (>3%) found in Lake Xiliang. Zonation was determined by CONISS (constrained incremental sums of squares) clustering.

Figure S4. Comparison of diatom reconstructions from sedimentary DNA and subfossil records. Smoothed trends were fitted using generalized additive models (GAMs). Diatom fossil data included only samples from the 1–35 cm interval.

Figure S5. Relationship between phytoplankton community dissimilarity (Bray–Curtis) and temporal distance.
